# Use of *Azospirillum baldaniorum* cells in quercetin detection

**DOI:** 10.5599/admet.1661

**Published:** 2023-03-21

**Authors:** Matvey V. Kanevskiy, Irina S. Kosheleva, Vladislav O. Menukhov, Elizaveta S. Zhdanova, Svetlana V. Borisova, Gennady L. Burygin, Svetlana A. Konnova, Victor D. Bunin, Olga I. Guliy

**Affiliations:** 1 Chernyshevsky Saratov State University, Saratov 410012, Russia; 2 Institute of Biochemistry and Physiology of Plants and Microorganisms – Subdivision of the Federal State Budgetary Research Institution Saratov Federal Scientific Center of the Russian Academy of Sciences (IBPPM RAS), Saratov 410049, Russia; 3 EloSystems GbR, Germany

**Keywords:** Flavonoids, quercetin, *Azospirillum*, electro-optical analysis, impedance

## Abstract

The possibility of detection and determination of flavonoids by using microbial cells was shown for the first time using the quercetin - *Azospirillum baldaniorum* Sp245 model system. The activity of the flavonoids quercetin, rutin and naringenin toward *A. baldaniorum* Sp245 was evaluated. It was found that when the quercetin concentration ranged from 50 to 100 μM, the number of bacterial cells decreased. Rutin and naringenin did not affect bacterial numbers. Quercetin at 100 μM increased bacterial impedance by 60 %. Under the effect of quercetin, the magnitude of the electro-optical signal from cells decreased by 75 %, as compared with the no-quercetin control. Our data show the possibility of developing sensor-based systems for the detection and determination of flavonoids.

## Introduction

Quercetin is one of the most widespread and studied flavonoids plant secondary metabolites that are actively used by humans owing to their high biological activity. The beneficial effect of flavonoids has been repeatedly proven in the treatment of a number of diseases, including cancer, Alzheimer’s disease, and atherosclerosis [1]. Flavonoids are indispensable in various pharmaceutical, medicinal, and cosmetic products, and they are used as antioxidants, angio- and cardioprotective drugs, antiinflammatory agents, antidiabetic and anticancer drugs, and so on [2-7].

The total flavonoid content is a very important indicator of the quality of plant materials, which is associated with the overall antioxidant activity of flavonoids. Because of the growing need for natural antioxidants, there is an increasing need to devise new flavonoid-detection methods and prepare standards for flavonoid determination [8].

The simplest way to detect and determine flavonoids is to use a spectrophotometric method based on the color reaction of flavonoids with aluminum chloride [9]. This method is recommended by the European Pharmacopoeia [8] because it is highly sensitive (up to 10 ng/ml), widely available, and does not require complex sample preparation. However, it is more suitable for determining the total content of phenolic compounds because individual flavonoids in a mixture are difficult to identify owing to the similarity of their spectra [10-12].

The most common methods for fractionating and detecting flavonoids in plant extracts are thin-layer chromatography (TLC) and high-performance liquid chromatography (HPLC). TLC is mainly used for the primary rapid analysis of the composition of isolated substances and is ineffective at separating mixtures of flavonoids [13]. HPLC quickly and effectively separates mixtures of flavonoids and their derivatives (O-methylated and glycosylated forms) and is highly sensitive (up to 1 ng/ml), but it requires expensive equipment and a wide variety of highly purified standards [14,15]. The use of solid-liquid extraction, solid-phase extraction, and ultrahigh-performance liquid chromatography coupled with tandem mass spectrometry strongly expands the possibilities of HPLC and makes it the most informative method for the characterization of the flavonoid structures in a mixture. The weak point, however, is the need for complex and expensive equipment and for highly trained operators [16,17]. For the analysis of flavonoids, capillary electrophoresis is also used, which is highly effective at separating substances [18,19] and is highly sensitive (up to 0.3 μg/ml). However, depending on the expected flavonoid composition in the extract, it becomes necessary to vary buffer composition, which limits the applicability of this method.

Few data exist on the use of bacteria-based biosensors for the detection and determination of flavonoids. In particular, Siedler *et al.* [20] showed that the introduction of special gene constructs makes it possible to use *E. coli* cells to detect flavonoids from 100 μM onward. But for the operation of the sensor to be successful, it is necessary to first transform bacterial cells and then carry out the detection with fluorescence, which also makes the method more expensive and labor-intensive.

Despite the many existing methods for the detection and determination of flavonoids, it is very important to search for new alternative and universal methods for their determination in extracts. A promising approach to detecting various compounds can be one that is based on the analysis of microbial cell responses. For example, Velichko *et al.* [21] showed that a decrease in the polarizability tensor γ of *Herbaspirillum lusitanum* cells is directly proportional to the content of sodium dodecyl sulfate in the medium in the range 0.01–2 mg/l.

Microorganisms can be used as a simple and fast-sensing element in the determination of the flavonoid of interest. In particular, nonpathogenic rhizobacteria of the genus *Azospirillum* can be used in biosensors because flavonoids inhibit the growth of *azospirilla* and change the physicochemical properties and composition of their surface glycopolymers [22,23].

Here, the possibility of detecting and determining quercetin when acting on *Azospirillum baldaniorum* Sp245 was evaluated by the changes in the physicochemical characteristics of bacteria.

## Experimental

### Bacteria and culture conditions

*A. baldaniorum* Sp245 (IBPPM 219) [24,25] was from the Collection of Rhizosphere Microorganisms, Institute of Biochemistry and Physiology of Plants and Microorganisms, Russian Academy of Sciences (IBPPM RAS) (http://collection.ibppm.ru). Bacteria were grown in an iron–free liquid malate medium [26] with constant stirring on a shaker at 30 °C for 24 h.

### Flavonoids

Quercetin (≥98 %; DIAEM, Russia), rutin (≥94 %; Sigma–Aldrich, USA), naringenin (≥95 %; Sigma–Aldrich; USA) were added to the medium as a solution in dimethyl sulfoxide (DMSO) to final concentrations 50, 100, and 200 μM. The content of DMSO in the medium was 1 vol.%. The inoculum was added to the medium to *A*_600_ = 0.09–0.11.

### Phase-contrast microscopy

Both control and flavonoid-incubated samples were subjected to phase-contrast microscopy (magnification, 40×) on a Leica LMD 7000 laser dissector (Leica Microsystems, Germany). The microscopy was done at the Simbioz Center for the Collective Use of Research Equipment (IBPPM RAS, Saratov). Samples were stained with aqueous 1 % crystal violet, as described in [27]. Microbial cells were placed on a coverslip, moistened with 1 % crystal violet solution, and incubated at room temperature for 30 min. The stain was then removed, and the samples were carefully washed with a phosphate buffer until the washing solution was completely clear. The stained biofilms were dried at 50 °C and subjected to phase-contrast microscopy.

### Measurement of orientational spectra of bacterial suspensions

Measurements were made with an ELUS EO analyzer (EloSystems GbR, Germany), as described in [28]. The measuring conditions were: electric field strength, 89.4 V/cm; light wavelength, 670 nm (relative to vacuum); field application time, 4.5 s. Before study, the bacteria were washed by double centrifugation at 2800 g for 5 min in distilled water (conductivity, 1.8 μS/cm). To remove cellular aggregates, we centrifuged the cell suspension again at 110 × g for 1 min and used the suspension that remained in the supernatant liquid for further work. The absorbance (*A*_670_) of the suspension was adjusted from 0.4 to 0.42. The orienting-field frequencies were 50, 100, 200, 300, 400, 500, 700, 1000, 2000, and 3000 kHz.

Orientational spectra were presented as the frequency dependence of the difference (*δA*) between the absorbance values of cell suspensions. This difference was measured during the preparation of a beam of unpolarized light along and across the orienting-field direction and was normalized to the absorbance value measured for randomly oriented cells [29,30].

**Figure 1.**
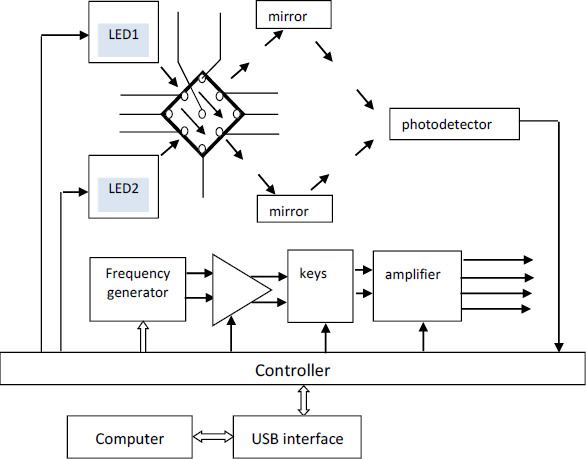
General block diagram of the EO analyzer. The photometric scheme is made according to a two-beam scheme. Two orthogonal light beams from LED1 and LED2 alternately pass through the EO cell. Then, by using a system of mirrors, the beams are focused on a photodetector. The EO cell contains nine electrodes, which are connected to an electric field generator. The electric field generator contains a frequency synthesizer with two antiphase outputs, a two-channel variable gain amplifier, a switch, and a power amplifier. The outputs of the four-channel power amplifier are connected to the groups of electrodes of the EO cell

Optical sensor systems are based on the effects of an electric field on cells suspended in an aqueous medium; i.e., they are based on the use of particle polarizability in an electric field and the measurement of the optical manifestation of the polarizability results [Kerr effect/electro-optical effect (EO)]. When an electric field is applied to a cell suspension, the cellular structures become polarized and, as a result, the cells acquire an induced cellular dipole moment. The physical manifestation of this effect is the electrical orientation of cells, which manifests itself as a transition of cells to an oriented state. The phenomenon of electric-field orientation is due to the effect of an external electric field on the dipole moment of cells. Polarization arises when a field is applied and decreases when it is removed - not instantly but after some finite time, called the relaxation time [29,30]. Figure 1 shows the general scheme for the EO analyzer.

### Impedance dispersion analysis

**Figure 2.**
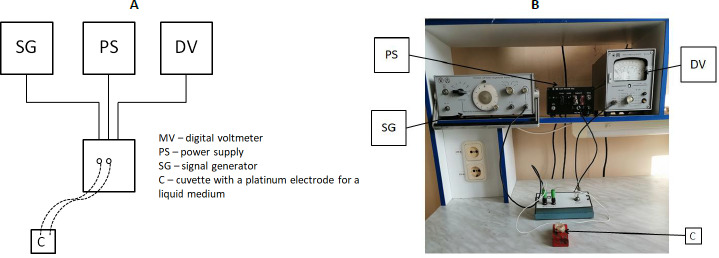
A) General scheme and B) appearance of the installation for impedance dispersion analysis

Figure 2 shows the general scheme and outside appearance of the installation used for impedance dispersion analysis. The container for impedancemetry was a 2-ml plastic container in which platinum electrodes were mounted at a distance of 1 cm from each other. Before the experiment, a control was done in which the resistance of the NaCl solution (9 g/l) was measured in the entire frequency range. The obtained value was subtracted from the experimental one to level the effect of the washing solution on impedance.

Bacteria were spun down by centrifugation (10,000 g, 5 min) and washed free of the medium twice with NaCl (9 g/l), after which the culture impedance was measured. Impedance dispersion was examined by using an installation consisting of a G6-27 signal generator, a V7-27 A/1 digital voltmeter, a K762 power supply unit, and a measuring cuvette (Russia).

A sample was placed in the measuring cell, and the incoming voltage (*U*_in_) (voltage in the circuit before the electric current passes through the sample) was measured. Next, the voltage in the circuit was measured after the current had passed through the sample (*U*_out_). For the measuring cell, the exemplary resistance *R*_0_ was calculated and found to be 4.99 kOhm (constant). In accordance with Ohm’s law, a formula was derived for calculating the impedance (1) because the voltage of the alternating current is proportional to its resistance.

The *U*_in_ and *U*_out_ were measured at 10^1^, 10^2^, 10^3^, 10^4^, 10^5^, and 10^6^ Hz, after which the resistance value was found from the formula:


(1)

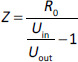



where *U*_in_ is the input voltage, *U*_out_ is the output voltage, and *R*_0_ is 4.99 kOhm.

Additionally, the polarization coefficient (PC) was calculated as an indicator of the ability of charged particles to move in the dielectric under the influence of an electric field. The PC was calculated by evaluating the steepness of the impedance dispersion curve and the cell viability. Intact cells have high resistance at low electric-current frequencies because the membrane acts as a capacitor. Membrane damage leads to a decrease in the capacitive component of impedance; accordingly, cell viability decreases and the impedance dispersion curve becomes flatter. The flavonoid-affected PC of the *Azospirillum* cells was also calculated. The PC [31] is the ratio between the impedance indicators at two frequencies, 10 kHz and 1 MHz.

### EPS extraction

For isolating extracellular polysaccharides (EPSs), the cells were pelleted by centrifugation at 10,000 g for 30 min. Three volumes of chilled ethanol were added to cold cell-free culture liquid, and the mixture was left to stand at 4 °C for 24 h [32]. The precipitated EPSs were separated by centrifugation (10,000g, 30 min), suspended, dialyzed against distilled water for 24 h, concentrated on a rotary evaporator, and lyophilized.

### Colony-forming-unit (CFU) counting

Colonies formed from individual viable cells after growth with the flavonoids were counted by the standard method of plating on solid nutrient media [33]. The control was the colonies grown without the flavonoids.

### Gas-liquid chromatography (GLC)

Samples were hydrolyzed with 2 M CF_3_COOH (120 °C, 2 h), reduced with NaBH_4_, and acetylated [34]. EPS monosaccharide composition was examined by GLC of polyol acetates on a Hewlett-Packard 5890 chromatograph.

### Antibodies

Polyclonal rabbit antibodies were prepared to glutaraldehyde-treated *A. baldaniorum* Sp245 cells, as described [35].

### Statistics

For each series of experiments, at least five repetitions were done. Data were analyzed with Microsoft Excel 2010 and with standard statistical processing methods.

## Results and Discussion

### CFU counting

**Figure 3.**
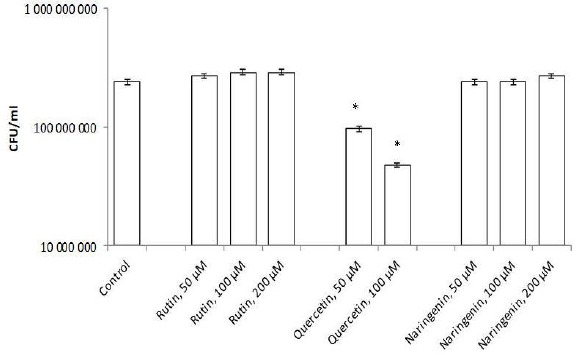
CFU of *A. baldaniorum* Sp245 grown with flavonoids. *Significant differences for P<0.01

Flavonoids can be divided into subclasses, including chalcones, flavones, flavonols, flavanones, and isoflavones. Quercetin is one of the first isolated and best-studied flavonoids; therefore, we investigated its effect on the rhizospheric bacterium *A. baldaniorum* Sp245. For this purpose, bacteria were grown with quercetin (concentrations 50, 100, and 200 μM) and postincubation changes in CFU were analyzed. The quercetin concentrations had been chosen on the basis of previous studies [23,30]. Figure 3 shows that quercetin at 50 and 100 μM inhibited culture growth, decreasing the cell number by 10 times (9.7×10^7^ and 4.8×10^7^ CFU/ml, respectively), as compared with the control (2.4 × 10^8^ CFU/ml). At 200 μM, quercetin precipitated, precluding further studies with this concentration.

**Figure 4.**
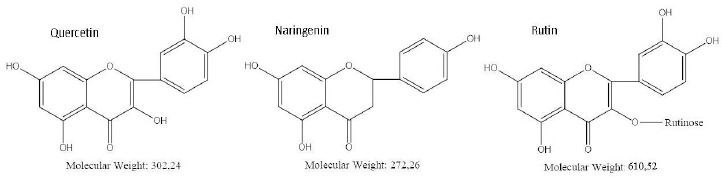
Structures of the flavonoids used.

To evaluate the specificity of the quercetin effect on *A. baldaniorum* Sp245, we also made studies with rutin and naringenin, whose structural formulas are similar to quercetin's (Figure 4). These flavonoids belong to different subclasses and, therefore, differ in hydrophobicity and degree of glycosylation [36,37]. The experimental procedure was the same as that used with quercetin. CFU counts showed neither rutin nor naringenin affected culture growth (Figure 3).

Quercetin has high biological activity, including bacteriostatic activity [38-40]. Importantly, quercetin and naringenin are aglycone forms of flavonoids; however, the addition of naringenin did not inhibit culture growth. Rutin, a glycoside of quercetin, did not affect the viability of *A. baldaniorum* Sp245 either. These results can be explained in terms of the chemical structure of the flavonoids used. According to the literature data [2], the antimicrobial activity of flavonoids is associated with the presence and number of hydroxyl groups in their structure. Unlike quercetin, naringenin does not have hydroxyl groups at positions 3 and 3', whereas in rutin, the hydroxyl group at position 3 is involved in the formation of a glycosidic bond with the rutinose residue (Figure 4).

### Phase-contrast microscopy

To confirm the inhibition of *A. baldaniorum* cell growth by quercetin at 50 and 100 μM, we used phase-contrast light microscopy. This kind of microscopy was chosen because the Leica LMD 7000 system makes it possible to identify relevant cells and ensures noncontact and contaminant-free isolation of individual cells. The high numerical aperture of the lens objectives and the short laser wavelength provide high-resolution images along the optical and transverse directions. This microscopy was used previously to evaluate the effect of antibacterial inhibitors on bacteria [41].

**Figure 5.**
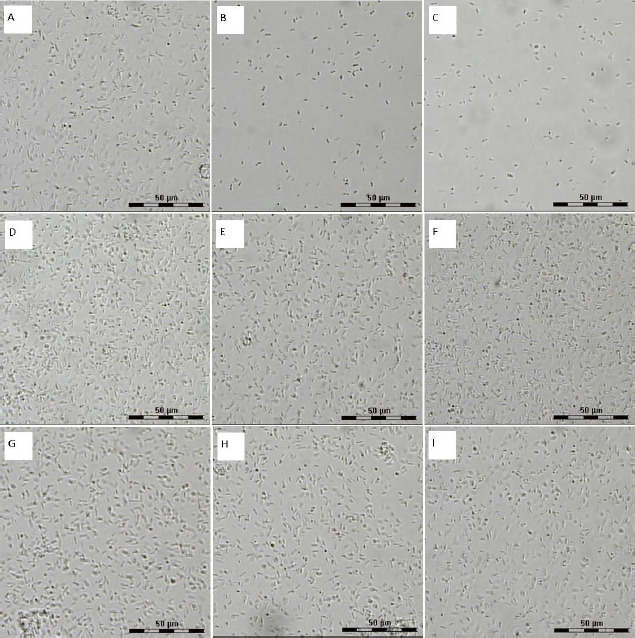
Light microscopy of *A. baldaniorum* Sp245. A) Control culture. Cultures grown with flavonoids: B) quercetin, 50 μM; C) quercetin, 100 μM; D) rutin, 50 μM; E) rutin, 100 μM; F) rutin, 200 μM; G) naringenin, 50 μM; H) naringenin, 100 μM; I) naringenin, 200 μM. The ruler size is 50 μm

Figure 5 shows the images for cell suspensions unexposed (Figure 5 A) and exposed to flavonoids (concentrations 50, 100, and 200 μM) (Figure 5 B-I). Neither naringenin nor rutin (Figure 5 D-I) affected the change in the cell number, as compared with the control (Figure 3 A). However, with increasing quercetin concentration (Figure 5 B-C), the number of cells in the field of view decreased compared to the control. This finding is consistent with the postexposure CFU counts.

### Impedancemetry of Azospirillum cells

**Figure 6.**
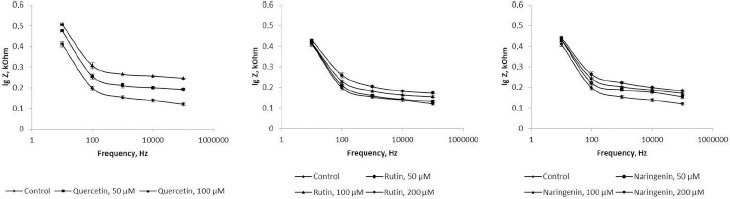
Impedance of *A. baldaniorum* Sp245 grown with A) quercetin, B) rutin, and C) naringenin

**Figure 7.**
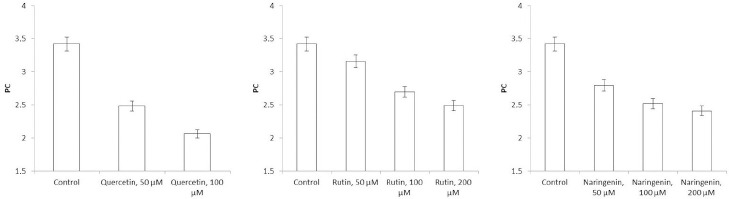
Polarization coefficients for *A. baldaniorum* Sp245 grown with **A)** quercetin, **B)** rutin, and **C)** naringenin

Because flavonoids can interact with the bacterial surface and change the barrier properties of the cell wall, impedancemetry was used to evaluate the effect of the flavonoids on *A. baldaniorum*. By impedancemetry, one can clearly show cell-resistance changes that occur under the influence of external conditions. The greatest influence on the impedance of *A. baldaniorum* Sp245 was exerted by quercetin at 100 μM (Figure 6 A). At high frequencies, the impedance value was greater than the control one by 2.1 times. The explanation for the sharp increase in bacterial ohmic part of resistance could be that high concentrations of quercetin in the growth medium stimulate the cell to stabilize the membrane. However, the PC (Figure 7 A) decreased with increasing flavonoid concentration and reached its minimum (60 %) at 100 μM, as compared with the control.

The change in the impedance of the cells incubated with rutin and naringenin (Figure 6 B and 6 C) was significantly less (by 43 and 51 %, respectively). The PC also decreased with increasing concentrations of flavonoids, but even at the maximum (200 μM) rutin and naringenin concentration, it did not decrease as much as it did with quercetin, equaling 73 and 71 %, respectively (Figure 7 B and 7 C).

In electrical engineering, impedance is the opposition to an alternating current presented by the combined effect of resistance and reactance in a circuit. For living objects, impedance is the sum of the resistance of the membranes (capacitive component) and that of the internal environment—the cytoplasm (ohmic component). The impedance of the internal contents depends only on the chemical composition of the cytoplasm and does not change depending on the frequency of the electric current. The impedance of the membranes will decrease with increasing frequency; at high frequencies, the impedance will be represented only by the ohmic component. Depending on the state of the membrane (intact or damaged), the value of the capacitive component will change, and hence the impedance as a whole will change, too. In our work, the influence of the external environment on impedance (ohmic part of resistance+reactance) was excluded because we examined the impedance of the cells washed from the nutrient medium. Figure 6 shows that the presence of quercetin in the growth medium causes changes in both capacitive and ohmic components of impedance. Because it is assumed that the effect of flavonoids on bacteria results in changes in the cell membranes, we considered only the change in the capacitive component of impedance.

### EO analysis of Azospirillum cell suspensions

Because flavonoids may damage the cell surface of bacteria, we used EO analysis to confirm the results obtained. Previous studies have used EO analysis to evaluate changes in microbial cells' electrophysical and morphometric variables subjected to an electric field. The evaluations were done in real time and did not involve special sample preparation. During measurements, the effect of the electric field on the cells was minimal and did not lead to cell death, and the effect of the supporting medium on the measuring accuracy was insignificant [29,42].

**Figure 8.**
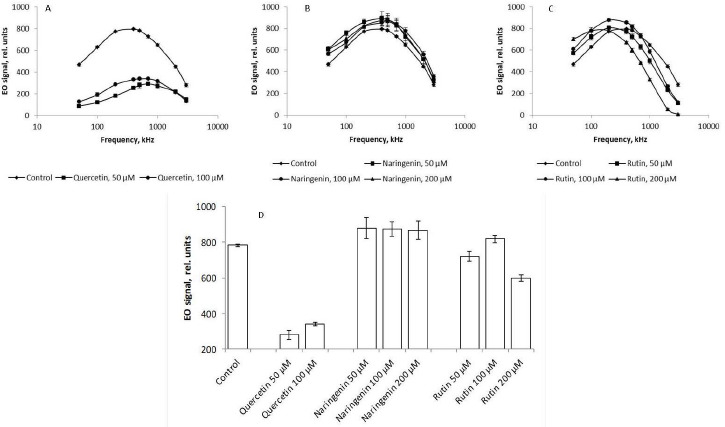
EO spectra of suspensions of *A. baldaniorum* Sp245 grown with A) quercetin, B) rutin, and C) naringenin. D) The magnitude of the EO signal at 500 kHz

The presence of quercetin in the growth medium lowered the EO signal up to 75 % across the range of frequencies used, as compared with the control (Figure 8 A). According to the literature, the cell surface charge depends on the integrity of cell membranes [43]. Quercetin-caused damage to bacterial membranes may decrease the cellular potential. According to the Goldman equation, damage to cell membranes is associated with an increase in their permeability and, consequently, passive transport between intra- and extracellular spaces decreases the transmembrane potential. In addition to the decrease in the cell charge as a result of quercetin-caused damage to the cell membranes, the membrane potentials of the cells decrease and the cells become dehydrated; this, ultimately, changes the EO signal (Figure 8 A).

The addition of naringenin led to a slight (no more than 3 %) change in the magnitude of the EO signal across the range of frequencies used (Figure 8 B), as compared with the control. The presence of rutin in the culture medium mediated an increase in the EO signal magnitude in the low-frequency region by 13 % (Figure 8 C). This indirectly indicates that the changes affected the polymers present on the cell surface. In the high-frequency range, on the contrary, the magnitude of the EO signal decreased by 20 %, as compared with the control, only with 200 μM rutin. These changes in the anisotropy of cell polarizability at 1000, 2000, and 3000 kHz, reflecting the state of the cytoplasm, are the earliest response to changes in the vital parameters of a bacterial culture under the effect of rutin and indicate a change in the physicochemical properties of the cell cytoplasm. For the convenience of presentation, Figure 8 D shows the change in the EO signal magnitude at an orienting-field frequency of 500 kHz.

**Figure 9.**
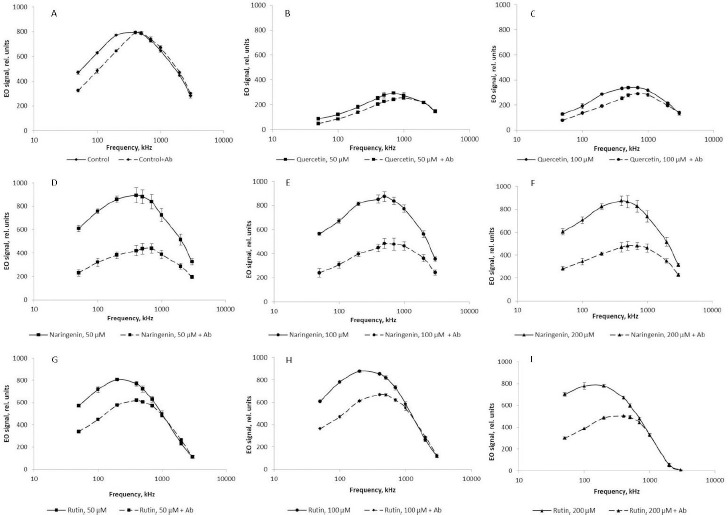
EO analysis of cell suspensions of *A. baldaniorum* Sp245. **A)** Control culture (antibodies to strain Sp245). Cultures grown with flavonoids: **B)** quercetin, 50 μM; **C)** quercetin, 100 μM; **D)** naringenin, 50 μM; **E)** naringenin, 100 μM; **F)** naringenin, 200 μM; **G)** rutin, 50 μM; **H)** rutin, 100 μM; **I)** rutin, 200 μM

At different orienting-field frequencies, different cell structures take part in the formation of an induced dipole, including cell surface structures, membrane biomolecules, and cytoplasm components [29]. At a low orienting-field frequency (below 200 kHz), an induced dipole is formed owing to the structures protruding into the extracellular space. The changes in the EO signal between 200 and 1000 kHz indicate a change in the membrane composition. The signal differences above 1000 kHz may indicate changes in the cytoplasm composition. Because we showed by impedancemetry and EO analysis that quercetin caused changes in the cell membrane surface, we made additional studies to confirm the preservation of the antigenic determinants characteristic of the *A. baldaniorum* Sp245 cell surface. The main component of the bacterial outer membrane is lipopolysaccharide (LPS); therefore, we used antibodies specific for the LPS of strain Sp245. It is known that changes in the EO signal in the low-frequency region (50–200 kHz) point to changes that have occurred on the surface of microbial cells [29,44]. Therefore, EO analysis was used to record interactions of the antibodies with the cell surface. Antibodies were added to the cell suspension to a final concentration of 6 μg/ml, and measurements were made 5 min after the start of cell incubation with the antibodies. The antibody concentration and exposure time were chosen on the basis of previous studies evaluating the effect of antibodies on bacteria [43]. The specific interaction of the antibodies with cells grown in the presence of quercetin (Figure 9 B, C), naringenin (Figure 9 D, E, F), and rutin (Figure 9 G, H, I) indicated that the antigenic determinants characteristic of Sp245 strain were preserved on the cell surface. However, the obtained results do not exclude the appearance of new structures on the cell surface.

### EPS extraction and monosaccharide composition analysis

Under the effect of flavonoids, the composition of *Azospirillum* polysaccharides undergoes changes [22,23]. Therefore, we isolated the EPSs of the cultures incubated with quercetin, naringenin, and rutin (each used at 100 μM) and examined their monosaccharide composition. GLC analysis showed the preservation of the qualitative composition of the EPS-constituting monosaccharides, in agreement with the data by EO analysis. However, the EPSs of the bacteria grown with rutin and quercetin showed an increased relative proportion of glucose (Table 1), possibly indicating the appearance of a new-composition polymer. A similar effect for LPS had previously been shown with this strain [45] as a result of the replacement of the carbon source in the nutrient medium. For other *Azospirillum* strains, modifications of the LPS composition and structure had also been found in bacteria grown with flavonoids and an increased proportion of rhamnose had been found [23]. Such changes may signal adaptation by bacteria transitioning from a free-living to a symbiotic lifestyle.

Today, much attention is paid to the search and use of plant materials in various industries, in particular as food, cosmetic, and nutritional supplements and as livestock feed and biomass sources. Phenolic compounds, ubiquitous in plants, are indispensable in the human diet and animal feed, mainly owing to their antioxidant properties. In the past 20 years, much attention has been paid to flavonoids, with most of the work being addressed to their potential positive effects and only a few aiming to further improve the existing methods of analysis. As recommended by the European Pharmacopoeia, flavonoids are isolated by extraction of plant material, usually with ethyl alcohol, methyl alcohol, or aqueous alcohols (most often, 70 % alcohol as an optimal extractant). This procedure is a lengthy one [8]. As can be seen from Table 2, the main methods used to determine flavonoids, including quercetin, take much time to complete.

Biosensors, which consist of a sensing element and a sensor for signal recording, hold promise for the detection and determination of flavonoids. An important point in the development of a bacteria-based sensor system is the selection of the sensing element. The use of *A. baldaniorum* Sp245 as the sensing element in the detection and determination of quercetin is very promising because, as shown in this work, quercetin specifically affects this bacterium and its response may indicate the presence of the flavonoid in the sample being tested. The obtained data may be useful in the development of a rapid method for the detection and determination of quercetin by using *A. baldaniorum* Sp245. The available literature contains almost no data on the use of bacteria as a sensing element in the detection and determination of flavonoids, including quercetin. The results of this work show the promise of EO analysis and impedancemetry, with *A. baldaniorum* Sp245 as the sensing element, for use in the detection of quercetin. The proposed approach can be used for the rapid detection of flavonoids and evaluation of the antioxidant activity of samples.

## Conclusions

Quercetin inhibits the viability of *A. baldaniorum* Sp245 from 50 μM onward. By contrast, rutin and naringenin do not affect *A. baldaniorum* viability. Quercetin at 100 μM has the greatest effect on the impedance of *A. baldaniorum* Sp245, as compared with rutin and naringenin. Under the effect of quercetin, the EO signal decreases by 75 %, as compared with the control. The quercetin-induced changes affect the polymers present on the cell surface. The interaction of specific antibodies with cells (after incubation with quercetin, naringenin, and rutin) indicates the preservation of characteristic antigenic determinants but does not exclude the appearance of new structures on the bacterial surface. We conclude that *A. baldaniorum* Sp245 is promising for use as a sensing element in the detection and determination of quercetin.

## Figures and Tables

**Table 1.** EPS monosaccharides composition in *A. baldaniorum* Sp245

Monosaccharide		Sample			
		Control	Quercetin (100 μM)	Rutin (100 μM)	Naringenin (100 μM)
Content, % of the sum of the polyol acetate peaks	Rhamnose	84.0±4,2	73±3.6	75±3.5	86±4.3
	Glucose	16.0±0.8	27±1.5	25±1.4	14±0.6

**Table 2.** Brief description of the methods used to determine flavonoids

Method	Flavonoid	Limit of detection	Analysis time, h	Reference
Physicochemical methods				
TLC	Quercetin Rutin Gallicacid	1 μg/ml 0.5 μg/ml 0.3 μg/ml	1–2	[13,46]
HPLC	Quercetin Morin Rutin	0.2ng/ml 0.4 μg/ml 0.2 μg/ml	1–2 h	[10,47-49]
HPLC–MS	Quercetin Rutin Apigenin Hesperedin	1 ng/ml 10 ng/ml 1.1 ng/ml 0.8 ng/ml	3–5 h	[16,50]
Capillary electrophoresis	Naringin Naringenin Quercetin Rutin Apigenin	0.4 μg/ml 0.3 μg/ml 0.3 μg/ml 0.5 μg/ml 0.5 μg/ml	1–2 h	[18,19]
Spectrophotometry	Total content of flavonoids	10 ng/ml	10–30 min	[10]
Biosensor-based methods				
Fluorimetric analysis of transformed bacterial cells	Quercetin	10 μg/ml	2–3 h (26-27 h*)	[20]
EO analysis, impedancemetry	Quercetin	10 μg/ml	1–2 h (25-26 h*)	This work

*allowance for culture growth
